# Dynamic Enhancer Methylation - A Previously Unrecognized Switch for Tissue-Type Plasminogen Activator Expression

**DOI:** 10.1371/journal.pone.0141805

**Published:** 2015-10-28

**Authors:** Mia Magnusson, Emma Xuchun Lu, Pia Larsson, Erik Ulfhammer, Niklas Bergh, Helena Carén, Sverker Jern

**Affiliations:** 1 Wallenberg Laboratory, Department of Molecular and Clinical Medicine, Institute of Medicine, Sahlgrenska Academy, University of Gothenburg, Gothenburg, Sweden; 2 Sahlgrenska Cancer Center, Department of Pathology, Institute of Biomedicine, Sahlgrenska Academy, University of Gothenburg, Gothenburg, Sweden; University of Bonn, Institute of Experimental Hematology and Transfusion Medicine, GERMANY

## Abstract

Tissue-type plasminogen activator (t-PA), which is synthesized in the endothelial cells lining the blood vessel walls, is a key player in the fibrinolytic system protecting the circulation against occluding thrombus formation. Although classical gene regulation has been quite extensively studied in order to understand the mechanisms behind t-PA regulation, epigenetics, including DNA methylation, still is a largely unexplored field. The aim of this study was to establish the methylation pattern in the t-PA promoter and enhancer in non-cultured compared to cultured human umbilical vein endothelial cells (HUVECs), and to simultaneously examine the level of t-PA gene expression. Bisulphite sequencing was used to evaluate the methylation status, and real-time RT-PCR to determine the gene expression level. While the t-PA promoter was stably unmethylated, we surprisingly observed a rapid reduction in the amount of methylation in the enhancer during cell culturing. This demethylation was in strong negative correlation with a pronounced (by a factor of approximately 25) increase in t-PA gene expression levels. In this study, we show that the methylation level in the t-PA enhancer appears to act as a previously unrecognized switch controlling t-PA expression. Our findings, which suggest that DNA methylation is quite dynamic, have implications also for the interpretation of cell culture experiments in general, as well as in a wider biological context.

## Introduction

Tissue-type plasminogen activator (t-PA) is a key component of the fibrinolytic system serving to protect against intra-vascular blood clot formation. The process of thrombus formation is continuously counteracted by t-PA, which in the presence of fibrin converts plasminogen to plasmin that breaks down the fibrin strands and leads to clot dissolution. t-PA is produced and stored in the endothelial cells lining the blood vessel walls. We have previously shown that the endothelial capacity for t-PA release is the main determinant of the efficiency of the local fibrinolytic response [1]. It is also known that this capacity is directly linked to the production rate of t-PA protein [2].

In 1995, a region several thousand bases upstream of the t-PA start site mediating the t-PA response to retinoic acid was identified by Bulens and coworkers [3]. Subsequently, a 900 bp minimal enhancer spanning between -7.1 and -8.0 kb relative the t-PA transcription start site was defined by progressive deletion analysis [4]. Since then, the t-PA enhancer has been shown to be crucial for basal gene expression levels [5]. In a previous study by our group, the single nucleotide polymorphism (SNP) -7,351 C/T was identified in the t-PA enhancer. The T allele, which breaks up a CpG dinucleotide constituting a Sp1 binding site, was found to convey a lower t-PA release *in vivo* [6] and, as expected, individuals carrying the T allele had an increased risk of suffering from myocardial infarctions [7, 8].

A better understanding of how the t-PA gene is regulated is crucial, as this could open up for the possibility of a pharmacological intervention in case of a sub-optimal t-PA response. Traditional cis/trans gene regulation has been quite extensively studied in relation to t-PA expression while epigenetics, referring to heritable changes in gene activity not caused by alterations in the DNA sequence itself, is a relatively unexplored field. The t-PA gene has previously been described to be sensitive to changes in histone acetylation status [9–11], which is one of the “classical” epigenetic modifications. We recently found that valproic acid, along with other clinically used histone deacetylase inhibitors (HDACi), strongly enhances t-PA gene expression [12, 13].

Another example of a classical epigenetic mechanism is DNA methylation, which occurs primarily on cytosine residues in the context of CpG dinucleotides and typically is associated with gene silencing. DNA methylation is established and maintained by DNA methyltransferases, and can be removed either passively or, as recently discovered, actively by DNA demethylases [14–16]. Traditionally, most DNA methylation studies have focused on CpG islands (regions with higher CpG density than ‘bulk’ DNA) in gene promoters. However, focus has recently been drawn to methylation alterations in other regions such as CpG island shores [17] and enhancers. One study found enhancer methylation to be the main determinant of gene transcription levels [18], and another study suggested that enhancer methylation, for some genes, determines cell type-specific gene expression [19]. In yet another study, it was discovered that both the enhancer and promoter of the OCT4 gene gained in methylation level after stimulation with retinoic acid (which also induced differentiation of the cell line used in the experiment) [20].

The t-PA promoter does not contain any CpG island, and there has, to our knowledge, only been one previous attempt to elucidate the methylation pattern in the t-PA promoter area in endothelial cells [10]. That study found cultured human umbilical vein endothelial cells (HUVECs) to be less methylated in the t-PA promoter compared to cell lines of various origins. The methylation status of the t-PA enhancer, however, has never been examined.

As epigenetics is a way for an organism or a cell to respond to the environment, we investigated if HUVECs modify their methylation pattern as a response to the environmental change they are subjected to when placed in culture. Because of the previously recognized importance of the enhancer in promoting t-PA expression, we analysed the methylation state of the t-PA enhancer along with the promoter. The aim of the present study thus was to establish the DNA methylation pattern in the t-PA enhancer and promoter in non-cultured HUVECs and cultured HUVECs at different passages in relation to t-PA expression.

## Materials and Methods

### Cell culture and experimental design

Fresh umbilical cords were obtained from the delivery ward at Sahlgrenska University Hospital, and human umbilical vein endothelial cells (HUVECs) were extracted by collagenase treatment [21]. In order to remove potentially contaminating cell types, primary non-cultured HUVECs were sorted with MACS magnetic beads (Miltenyi Biotec, Bergisch Gladbach, Germany) directed against the endothelial cell surface antigen CD105. HUVECs were grown in complete endothelial cell culture medium (EGM-2, Lonza, Basel, Switzerland), and sub-cultured by trypzination (Lonza) until passage 4. For DNA purification, a cell fraction was extracted from primary HUVECs before placed in culture, as well as from cultured HUVECs at passage 0 and 4. HUVECs cultured for mRNA analysis were harvested at corresponding points, as well as at passage 1, 2, and 3.

The investigation follows the principle of the Declaration of Helsinki for the use of human tissues. The use of cells from human umbilical cords was approved by the Regional Ethics Review Board of Gothenburg (no. 449–93). Verbal informed consent was obtained from the donors regarding the use of umbilical cord cells for research purposes. Given that the study material was non-identifiable, written consent was uncalled for and this consent procedure was approved by the ethical review board.

### DNA methylation analysis

DNA methylation was assessed by bisulphite conversion, a method that relies on the ability of sodium bisulphite to convert unmethylated cytosines to uracil (which will be replaced by thymine by the DNA polymerase), while methylated cytosines remain unaffected [22].

DNA from primary (non-cultured), passage 0, and passage 4 HUVECs was extracted using the QIAamp DNA Mini Kit (Qiagen, Hilden, Germany) according to the manufacturer’s instructions. 200–500 ng of DNA was subsequently bisulphite-converted with the EZ DNA methylation kit according to the protocol provided (Zymo Research Corporation, Irvine, USA).

We identified 20 CpG dinucleotides in the t-PA enhancer, 9 in the proximal promoter region, and 7 in the upstream promoter region. For PCR amplification of the enhancer region, nested primers specific for bisulphite-treated DNA were designed using the MethPrimer software (www.urogene.org). For the proximal promoter region, as well as for the region immediately upstream of the proximal promoter, nested primers published by Dunoyer-Geindre were used [10]. Primer sequences can be found in Table 1. 15–40 ng of the bisulphite-treated DNA was subjected to 35 cycles of PCR amplification, with annealing temperatures for the different primer pairs spanning between 49 and 55°C. Fragment sizes were verified on 2% agarose (Sigma-Aldrich, St. Louis, MO, USA) gels supplemented with GelRed Nucleic Acid Gel Stain (Biotium, Hayward, CA, USA). For each PCR run, an unmethylated and a methylated control sample (EpiTect control DNA, Qiagen) as well as 30/70, 50/50, and 70/30 mixtures of the two were co-amplified to generate a methylation standard curve.

**Table 1:** Sequences and positions for the nested bisulphite primers.

primer	sequence	position
t-PA enh. upstr. out fw	TATATATATTAGATGGTAATTTTAGAATGG	-8,104 to -7,784
t-PA enh. upstr. out rv	ATCTAAAAAACACAAAACTTCCTAT	
t-PA enh. upstr. in fw	GGTGGGTTTGTGTTATTGTTT	-8,075 to -7,798
t-PA enh. upstr. in rv	AAACTTCCTATCTAATTTTAATCACC	
t-PA enh. mid out fw	TGTTTATTTAGGGTTTTGTGTTTTG	-7,965 to -7,464
t-PA enh. mid out rv	TCTTACATCAATTCTAATTACCCAC	
t-PA enh. mid in fw	TTGTATATATAGGGAGATAGGATAA	-7,886 to -7,497
t-PA enh. mid in rv	ACATATACTCAAAATAACAAAAACA	
t-PA enh. out fw	AAAATGTTTGATTTAGGAAGGAGGT	-7,602 to -7,268
t-PA enh. out rv	AACAATAACCAAAACCAAATAAATACAA	
t-PA enh. in fw	GGAGGTGGGTTTTATGTAAATAGTG	-7,547 to -7,297
t-PA enh. in rv	TAATTCCTTCTAACCCCAAAATAAC	
t-PA prom.1 out fw	TTTGAAAAGGTGTTAGTAAG	-832 to -494
t-PA prom.1 out rv	ACCACTAAAAAAACAAAACC	
t-PA prom.1 in fw	TAAGGGAAATGGTTTGTTTA	-816 to -528
t-PA prom.1 in rv	CTACRATAAAAAATACCCCCATA	
t-PA prom.2 out fw	TTTGGGTTTATTTAAGGGGATGT	-688 to +45
t-PA prom.2 out rv	AAAAATTTTCTCTCCAACCCTAAAC	
t-PA prom.2 in fw	GAGGTTATTTATTGTAGTTTTGTATTTTAT	-646 to +7
t-PA prom.2 in rv	CAACTCTAAACTCCCCACAACTC	
t-PA prom.3 out fw	TTAGGATTTTAAAGGAAGATGATTTTTAA	-287 to +109
t-PA prom.3 out rv	AAAAAAACAAACCCCAAAATACAA	
t-PA prom.3 in fw	AAAGGAAGATGATTTTTAAGGTTTTATTT	-277 to +44
t-PA prom.3 in rv	AAAATTTTCTCTCCAACCCTAAACT	

The PCR products were sequenced in both forward and reverse directions at Genomics Core Facility at the University of Gothenburg, and at GATC Biotech (Constance, Germany). In the subsequent analysis, raw data from the Sanger sequencing reactions were used as previously described [10, 23]; however, instead of directly measuring the peak heights in the chromatogram, we related our samples to a standard curve with known ratios of methylated/unmethylated DNA. This was done in order to control for selective amplification of methylated or unmethylated template during the PCR reactions, but also because it bypassed the problem of varying peak heights in the sequencing trace (caused by the different flurochromes, coupled to the nucleotides, having different intensities). Semi-quantitative methylation levels (in ten steps) based on relative C and T peak heights in the sequence profiles were assigned after a visual comparison between the sample and the standard curve (which included the five reference points corresponding to 0%, 30%, 50%, 70%, and 100% methylation) (S1 Fig). The analysis of the sequencing traces was performed in the Applied Biosystems SeqScape Software 3 (Life Technologies, Carlsbad, CA, USA).

HUVECs from 11 subjects (ID 001–011) were analysed for enhancer methylation. Four of the subjects (ID 001–004) were analysed also for promoter methylation (while material from ID 005–011 instead was used for gene expression analysis).

The standard curve method was verified by pyrosequencing of two selected CpG sites in the t-PA enhancer (S2 Fig). The pyrosequencing primer sequences were as follows: Forward: GTGGGTAATTAGAATTGATGTAAGAGT, Reverse: Biotin-AATAACCCCAAAATCCCAAAC, Sequencing: TTTTTTTTAGGTTTGAGTGAT. The PCR reaction was run using the PyroMark PCR Kit (Qiagen) according to protocol, with an annealing temperature of 59°C. The sequencing was performed according to the PyroMark Q24 Advanced and PyroMark Q24 Advanced CpG Reagents Handbook (Qiagen), and run on a PyroMark Q24 system. The pyrosequencing assay was evaluated with a standard curve of methylated/unmethylated template in the same five reference points described above.

The positions are given in relation to the major t-PA transcription initiation site (TIS). All sequences and positions in this study are according to the Dec. 2013 Genome Reference Consortium GRCh38 assembly obtained from the UCSC Genome Browser database (http://genome.ucsc.edu/).

### Gene expression analysis

Total RNA was prepared using RNeasy Mini Kit (Qiagen) according to the manufacturer’s protocol, and genomic DNA was removed using RNAse free DNase (Qiagen). RNA was transcribed into cDNA using the High-capacity RNA-to-cDNA Kit (Life Technologies). mRNA levels were analysed with real-time RT-PCR on an Applied Biosystems 7500 Fast Real-Time PCR system using Taqman reagents from Life Technologies. t-PA was detected with Gene Expression Assay Hs00938315_m1 (Life Technologies). We analysed a set of commonly used reference genes, and glucuronidase beta (GUSB) was found to have the most stable gene expression in all cell culturing passages, and was therefore selected as endogenous internal standard. GUSB was detected with Gene Expression Assay Hs99999908_m1 (Life Technologies).

Twelve subjects were included in this analysis. Seven of the subjects (ID 005–011) were the same as in the enhancer methylation analysis. ID 005–011 were also used for a correlation study between change in t-PA gene expression and change in enhancer methylation.

### Statistical analysis

For statistical analysis of change in t-PA gene expression as a result of culturing time/passage number, a one-way ANOVA for repeated measures was used. For correlation analysis between methylation change and change in t-PA gene expression comparing primary and p.0 HUVECs, a Spearman’s correlation test was used (after rank transformation of the data).

## Results

### t-PA enhancer methylation declines during cell culturing

**Fig 1:**
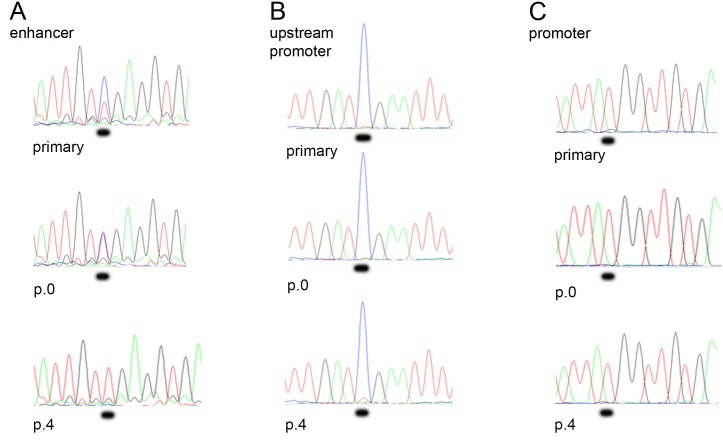
Gradual demethylation occurs in the enhancer but not in the promoter regions during cell culturing. Chromatogram showing the t-PA enhancer region in primary, passage 0, and passage 4 HUVECs sequenced in the (A) enhancer, (B) upstream promoter, and (C) proximal promoter regions. The position of the original cytosine residue is underlined. The blue peak corresponds to cytosine (methylated) and the red peak to thymine (unmethylated).

**Fig 2:**
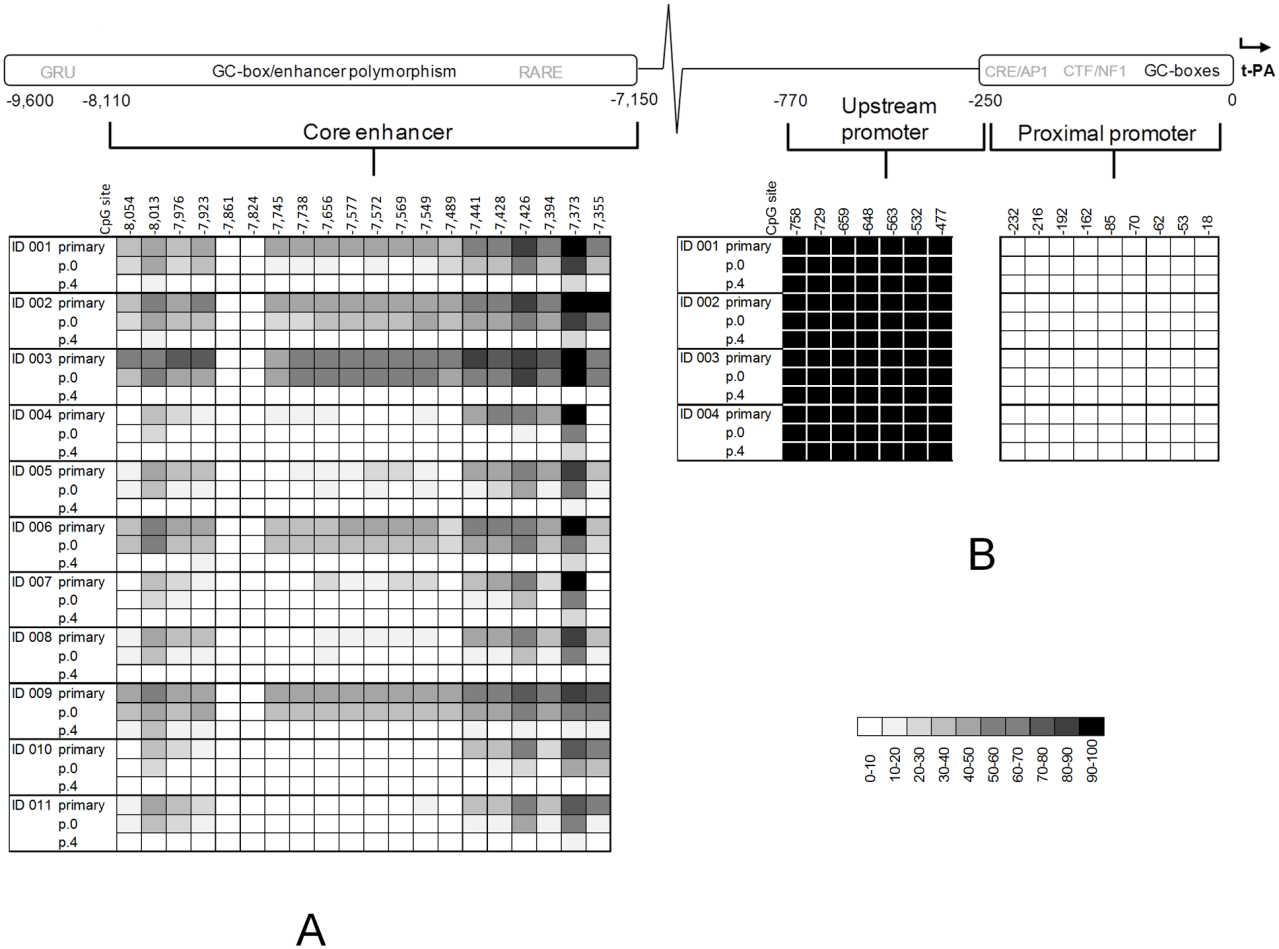
DNA methylation in t-PA enhancer, upstream promoter, and proximal promoter regions in primary, p.0, and p.4 HUVECs. (A) The methylation level in the t-PA enhancer in primary, passage 0, and passage 4 HUVECs from 11 subjects (ID 001–011) as determined by direct sequencing of bisulphite-treated PCR-amplified DNA. All the CpG dinucleotides in the core enhancer, stretching from -7,150 to -8,110, were included in the analysis. The most 3’ enhancer CpG site, -7,355, corresponds to the t-PA -7,351 C/T enhancer polymorphism (which, using updated genome assemblies, has been remapped to -7,355 relative the major TIS). (B) The methylation levels in the upstream promoter and promoter regions from four subjects (ID 001–004). The 9 CpG dinucleotides in the t-PA proximal promoter, stretching from TIS to -240, were analysed, along with the 7 CpG dinucleotides in the region immediately upstream of the promoter (from -240 to -760).

As an indication of the stability of the DNA methylation pattern in the t-PA enhancer and promoter, the levels of methylation in these regions were compared between primary (non-cultured), passage 0 (4–5 days in culture), and passage 4 (15–17 days in culture) HUVECs from the same subjects (Figs 1 and 2).

We found that a gradual but rapid demethylation occurred in the t-PA enhancer region as the cells were cultured (S1 Table).

In primary HUVECs, on average 17 of the 20 CpG sites were methylated (Fig 2A). The average total methylation level in the enhancer in primary HUVECs was 30–40%, ranging between approximately 20% (ID 010) and 60% (ID 003). Notably, the two CpG sites -7,824 and -7,861 were unmethylated in all subjects, while the CpG site -7,373 was at least 80% methylated in all subjects.

In p.0 HUVECs, 14 of the 20 enhancer CpG sites were methylated. The average total level of methylation in the enhancer of the p.0 HUVECs was approximately 20–30%, varying between less than 10% (ID 004) and around 50% (ID 003). Each of the 11 subjects included in the analysis displayed lower methylation levels compared to the primary cells, with methylation levels decreasing with between approximately 20% (ID 003) and 80% (ID 004).

In p.4 HUVECs, most of the enhancer CpG sites were unmethylated. However, at CpG site -7,373, some degree of methylation remained in the majority of the subjects.

### t-PA promoter and upstream promoter region methylation is stable during cell culturing

While the t-PA enhancer displayed decreasing methylation levels, this was not the case in the t-PA promoter (Fig 2B). We found that the t-PA promoter was unmethylated in primary HUVECs, and that it remained so as the cells were cultured.

The region immediately upstream of the t-PA promoter was included in the analyses as a control, as it should be of less importance for the expression of the t-PA gene. This region was found to be fully methylated in primary HUVECs. As the cells were cultured, the methylation level in this region remained unaffected.

### t-PA gene expression increases during cell culturing

**Fig 3:**
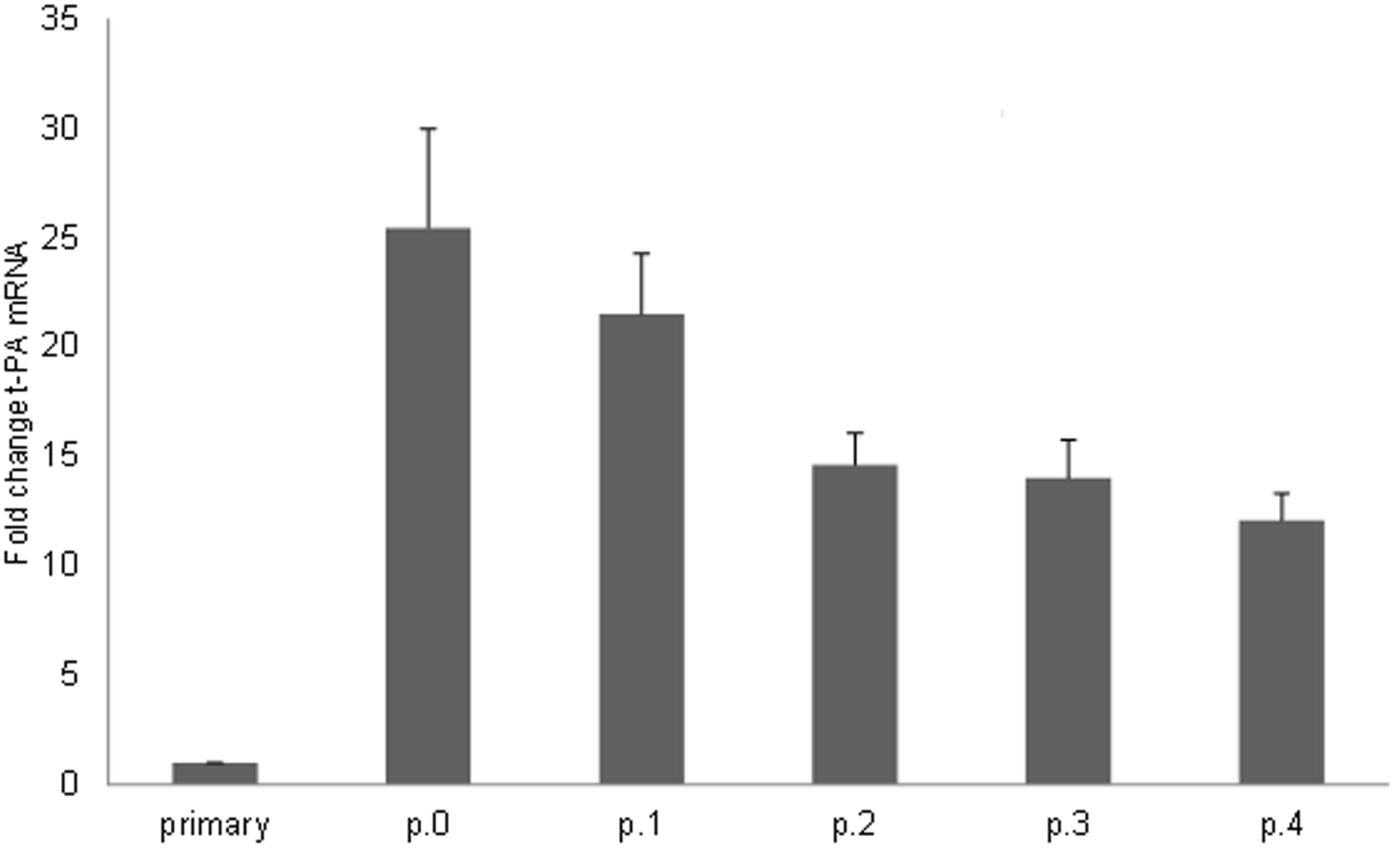
t-PA gene expression in primary and cultured HUVECs. Relative mRNA expression of tissue-type plasminogen activator (t-PA) in non-cultured HUVECs and p.0-4 HUVECs from the same subjects as determined by real-time RT-PCR. 7 of the 11 subjects from the methylation analysis (ID 005–011) were included also in this analysis (total n = 12). p<0,001 (one-way ANOVA).

**Fig 4:**
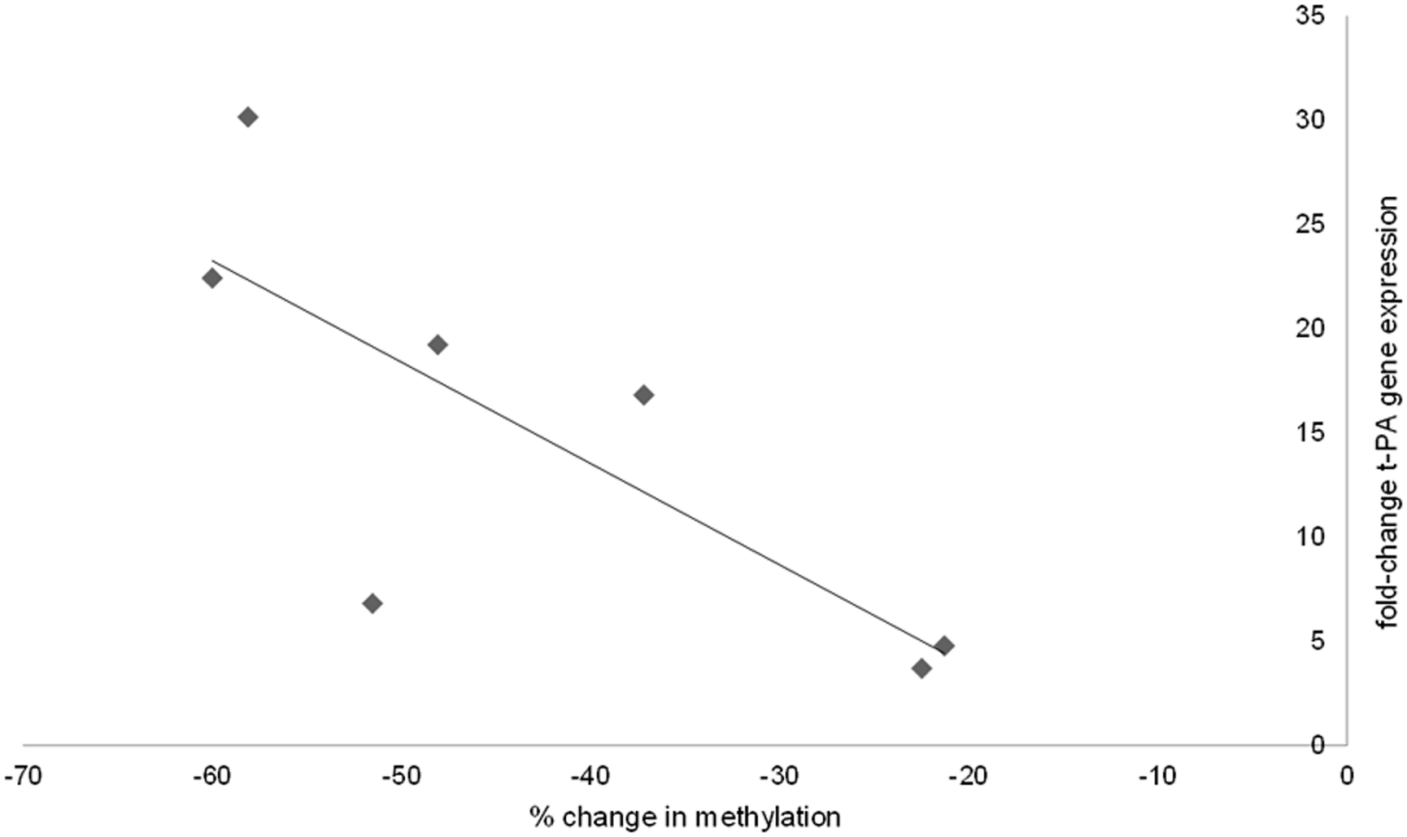
Correlation between decrease in methylation levels and increase in t-PA expression. A correlation analysis was performed on change in methylation and change in gene expression between primary and p.0 HUVECs. After rank transformation of the data, a Spearman’s correlation test gave a correlation coefficient of -0.86. The seven individuals (ID 005–011) for which both methylation and gene expression data were available were included in the analysis (n = 7, p<0.05). The figure depicts crude data.

To investigate if the altered methylation in the t-PA enhancer corresponded with a change in t-PA gene transcription, real-time RT-PCR of t-PA mRNA was performed on primary and passage 0 to 4 HUVECs (Fig 3). These experiments revealed that t-PA gene expression was increased by a factor of approximately 25 in passage 0 HUVECs compared to primary HUVECs. This was in strong negative correlation with the change in methylation level observed between primary and p.0 HUVECs (r = -0.86 and p<0.05 using a Spearman’s rank correlation test) (Fig 4). t-PA gene expression levels were elevated in all passages analysed compared to primary cells.

## Discussion

In this study, we found that the methylation level in the t-PA enhancer gradually but rapidly was reduced as HUVECs were cultured, and that this was strongly negatively correlated with an increase in t-PA gene expression.

We found primary HUVECs to be on average 30–40% methylated in the t-PA enhancer region. In passage 0 HUVECs, the average methylation level was reduced to around 20–30% and in passage 4 HUVECs, almost no trace of methylation could be detected. The t-PA promoter, on the other hand, was unmethylated in primary HUVECs and remained so as the cells were cultured. The region immediately upstream of the promoter, which should be of little importance for t-PA gene expression, was included in the analysis as a control. This region was heavily methylated in the primary cells; a pattern which remained throughout the cell culturing. To evaluate if there was an effect on t-PA gene expression, t-PA mRNA levels from primary to passage 4 HUVECs were quantified, revealing a pronounced increase (by a factor of approximately 25) in t-PA expression already in the passage 0 HUVECs. The decrease in the level of methylation was in strong negative correlation with the increase in gene expression.

Previously, promoter methylation in particular has been considered closely connected to gene expression (reviewed in [24]), and the two previous studies examining DNA methylation in relation to the t-PA gene have indeed evaluated only the promoter methylation level [10, 25]. Enhancer methylation has only recently gained attention. One study found altered gene expression to be more closely correlated with altered enhancer methylation than with altered promoter methylation [18]. That study suggested that, for some genes, enhancer methylation may serve as a main determinant of gene transcription levels.

The t-PA enhancer is well-established and known to be essential for gene expression, which we have confirmed in transfection experiments (where the enhancer induced a significantly higher gene expression compared to the promoter alone, S3 Fig). Of note, experiments with transgenic mice harbouring different lengths of the t-PA regulatory region fused to a lacZ reporter gene have revealed that the t-PA enhancer region seems to control tissue-specific expression of t-PA [26]. Given this established importance of the t-PA enhancer for gene expression levels, it is not surprising to find that t-PA may belong to the category of genes where the enhancer methylation governs gene expression.

DNA methylation has traditionally been perceived as a stable modification responsible for long-term repression of gene expression [27, 28]. However, recently, there have been reports of a more dynamic CpG methylation that can be affected by e.g. long-term culturing [29], and that, in pluripotent undifferentiated cells, can change depending on the culture condition [30]. Interestingly, the former study found these dynamic CpGs to be co-localized with transcription factor binding sites, and specifically with enhancers.

Still, to our knowledge, there are no previous studies where methylation levels have been compared between non-cultured cells and cultured cells at low passages. Instead, cells at higher passages are used as representatives of the primary cell type and the consensus view has indeed been that methylation changes are unlikely to have occurred in early passage cells [10]. In one study, it is stated that primary cell lines are suitable to use as models for understanding tissue-specific regulation of DNA methylation [31]. Surprisingly, our study reveals that cell culture may alter methylation levels faster than previously anticipated. Therefore, we believe that it cannot and should not be assumed that DNA methylation levels are stable even between cells at low passages. Thus, our results indicate that cultured cells may not always be a reliable model, especially when various aspects of gene expression are studied. In future studies, it is of importance to consider at which passage cultured cells are used. This should also be taken into consideration when reviewing previous studies.

As epigenetics is a way for a cell or an organism to respond to changes in the environment (reviewed in [32]), it is not completely unexpected to find altered DNA methylation patterns and changed t-PA gene expression levels in HUVECs that have been cultured. However, we do not know if it is the culturing *per se* that causes the switch. It could well be a biological phenomenon; t-PA is a proteolytic enzyme, and it may be beneficial for a cell to increase its t-PA expression when in a proliferative or migrating phase.

Ideally, a rescue experiment to remethylate the t-PA enhancer in cultured endothelial cells would give a more direct proof of a causal relationship between the change in t-PA enhancer methylation and gene expression. However, to specifically remethylate the t-PA enhancer is not possible with the methodologies available today. Even though we do have many pieces of the puzzle, like a strong correlation between DNA methylation and gene expression also on an individual level, in the future such an experiment will provide the final proof of a cause and effect relationship.

Whether the observed demethylation is passive or active in nature cannot clearly be elucidated from our study. As cells in culture grow and divide, it is difficult to distinguish between the two modes. Also, bisulphite sequencing, while considered the “gold standard” in methylation analysis [33], reveals the level of methylation but does not give additional information about possible modifications of the methyl group itself. One such modification is 5-hydroxymethylcytosine (5-hmC), which has been suggested to be involved in the process of demethylation. However, 5-hmC and the family of ten-eleven-translocation (TET) enzymes are proposed to be involved in both active and passive demethylation (reviewed in [34, 35]); thus, even if it was possible to get reliable measurements of 5-hmC levels and TET enzyme activity in the t-PA enhancer area, we would still not be certain about the nature of the demethylation.

One could perhaps argue that a fraction of HUVECs might have been unmethylated in the t-PA enhancer initially, and that this subpopulation was selected for during the culturing process. In that case, cell culturing would not have induced a demethylation event, but rather the selection of a certain subpopulation. This scenario, however, is highly unlikely as at least one of the enhancer CpG sites (-7,373) was completely methylated in the primary state in more than half of the individuals (Fig 2). If an unmethylated subpopulation still had existed in the primary cells, this population would not only have been too small to detect, but would also have been unable to grow to constitute as much as half of the total cell population after only a few days in culture (Fig 2A, ID 004, 006, and 007 at p.0).

Because the HUVECs have been subjected to the stress of a delivery that may take many hours and that will cause changes to the blood flow and in the supply of for example nutrients and oxygen to the endothelial cells, we cannot be sure that that the primary HUVECs actually represent the “natural”, or original, state. Because, as we demonstrate in this study, a methylation change can be fairly rapid, it is possible that a shift had already occurred by the time that we obtained the umbilical cords. Nonetheless, our study clearly demonstrates that methylation changes occur quite fast, and that gene expression levels quickly can be altered simultaneously.

Primary, passage 0, and passage 4 HUVECs were analysed in this study. We chose to study passage 0 HUVECs to evaluate if an alteration in the methylation status could be detected after such a short time in culture. Passage 4 HUVECs were included partly because the rapid change in methylation levels that we had indications of was likely to be established by that time, and partly because this is a passage in which many experiments are performed.

In this study, standard bisulphite sequencing was the method of choice as, according to Wilson *et al*, bisulphite sequencing of PCR fragments containing less than 22 CpG sites will not cause biased amplification of methylated or unmethylated template (which can occur due to differences in annealing temperature in amplicons with many CpG sites) [36]. However, because the method is based on Sanger sequencing, there are two major issues that have to be considered when assessing methylation levels. Firstly, the fluorochromes attached to the different nucleotides have different intensities leading to varying peak heights. Secondly, and perhaps more importantly, when one of the bases is underrepresented in the reaction (like in bisulphite sequencing) there will be an overcompensation (inherent to the sequencing program) of the signal strength of that particular base. This can be seen in the standard curve depicted in S1 Fig; if the differences in fluorochrome signal intensity or overcompensation did not occur, the C and T peaks in the variable position in the 50% methylated sample would have been expected to be of equal heights. This is, however, clearly not the case—the C peak is higher than the T peak. Thus, a measurement of exact peak heights as described earlier [10, 23] might have resulted in an incorrect description of methylation levels in this study. In order to avoid these potential problems, we chose to assess methylation levels in our samples by comparing them to a standard curve with known ratios of methylated/unmethylated DNA. This approach resulted in a semi-quantitative measurement of methylation, which we could verify with pyrosequencing to be very accurate (S2 Fig).

In summary, we found the t-PA proximal promoter to be unmethylated, while the directly adjacent upstream promoter region was fully methylated. This pattern was static, as it was present in primary HUVECs and did not change during cell culturing. Surprisingly, the methylation pattern in the t-PA enhancer behaved differently; here, the level of methylation was quickly decreased during cell culturing. We found the shift in enhancer methylation to be in strong negative correlation with t-PA gene expression levels, and thus it may constitute a way for the cell to dynamically respond to the environment. We suggest that an unmethylated t-PA promoter most likely is a prerequisite for active gene expression, while the enhancer methylation is dynamic, acting as a switch which allows the cell to regulate the level of gene expression as a response to the external environment.

Our findings are novel in two ways. Firstly, our results indicate that DNA methylation during cell culturing may be a more dynamic modification than previously recognized, as high methylation levels in the t-PA gene can be completely and stably erased after just a few days. Secondly, we found that the demethylation event is specific, as it occurs only in the t-PA enhancer and not in the promoter nor in the region immediately upstream of the promoter. We therefore hypothesize that methylation of the t-PA enhancer acts as a previously unrecognized switch that can be used to turn on t-PA transcription in response to external stimuli.

## Supporting Information

S1 Fig Illustration of the methylation standard curve. The cytosine of the original CpG site is underlined. The C peak is blue, and the T peak is red.
(TIF)

S2 Fig Validation by pyrosequencing. The pyrosequencing assay was designed to cover two CpG sites (-7,394 and -7,373), which were considered of interest as they differed from each other, as well as showed markedly decreased methylation levels over the three passages. The primer sequences were as follows: Forward: GTGGGTAATTAGAATTGATGTAAGAGT, Reverse: Biotin-AATAACCCCAAAATCCCAAAC, Sequencing: TTTTTTTTAGGTTTGAGTGAT. The PCR reaction was run using the PyroMark PCR Kit (Qiagen) according to protocol, with an annealing temperature of 59°C. The sequencing was performed according to the PyroMark Q24 Advanced and PyroMark Q24 Advanced CpG Reagents Handbook (Qiagen), and run on a PyroMark Q24 system. Four individuals from the original study (ID 003, 005, 008, and 009) were reanalyzed using pyrosequencing. The methylation level at each site varied on average 2.5% between the two methods (bisulphite sequencing with a standard curve, and pyrosequencing), and the standard deviation of the difference was 2.8%.
(TIF)

S3 Fig Transfection experiment—the t-PA enhancer strongly increases gene activity. The t-PA proximal promoter and the t-PA enhancer was PCR amplified and cloned into the XhoI/HindIII sites of a firefly luciferase reporter vector (pGL4-Luc, Promega), either each separate, or combined. All constructs were verified by sequencing. Subsequently, the constructs were co-transfected into HT-1080 cells together with the renilla luciferase control vector hRluc/SV40. The luciferase luminescence was measured and normalised to the renilla signal. Fold change of normalised signal is displayed in the figure. Constructs including both promoter and enhancer showed a 9-fold increase in reporter activity compared to constructs containing the promoter alone (p < 0.001, student’s t-test).
(TIF)

S1 Table Enhancer methylation average, enhancer methylation change, and fold change of t-PA expression. The methylation average and the change in methylation in the t-PA enhancer (in percent) for all 11 individuals, as well as fold change of t-PA expression (normalised to GUSB) for seven of the individuals (ID005-011).
(DOCX)
