# Supplementary figures and images for: Dynamic Enhancer Methylation - A Previously Unrecognized Switch for Tissue-Type Plasminogen Activator Expression
Source: PLoS One. 2015 Oct 28;10(10):e0141805. doi: 10.1371/journal.pone.0141805 (PMC4625093; doi:10.1371/journal.pone.0141805)

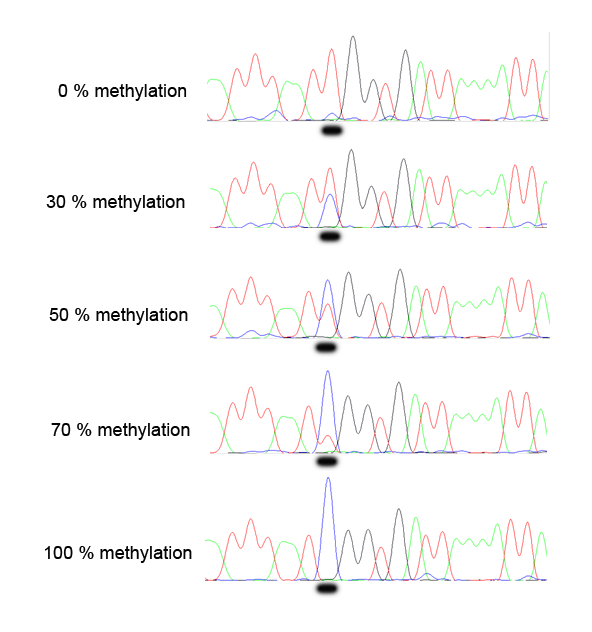

Supplement: S1 Fig — The cytosine of the original CpG site is underlined. The C peak is blue, and the T peak is red. (TIF) [file pone.0141805.s001.tif]

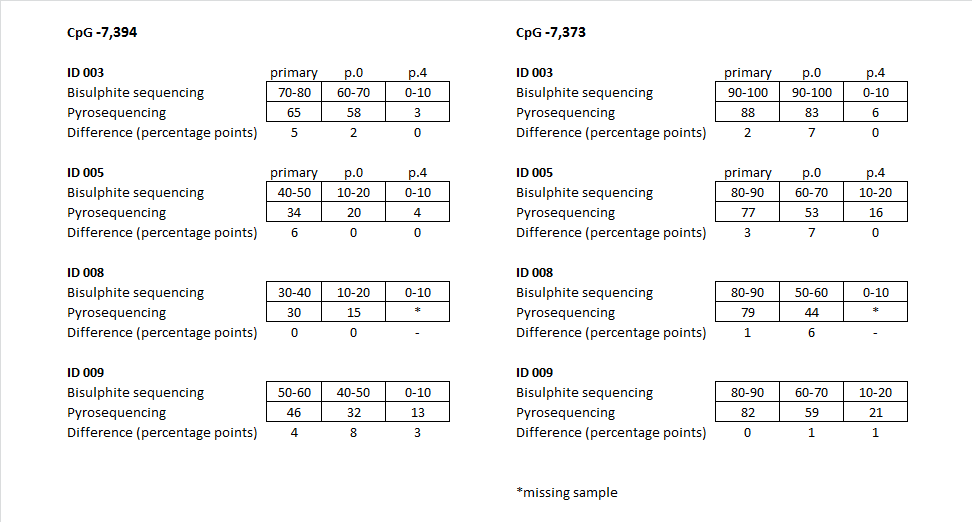

Supplement: S2 Fig — The pyrosequencing assay was designed to cover two CpG sites (-7,394 and -7,373), which were considered of interest as they differed from each other, as well as showed markedly decreased methylation levels over the three passages. The primer sequences were as follows: Forward: GTGGGTAATTAGAATTGATGTAAGAGT, Reverse: Biotin-AATAACCCCAAAATCCCAAAC, Sequencing: TTTTTTTTAGGTTTGAGTGAT. The PCR reaction was run using the PyroMark PCR Kit (Qiagen) according to protocol, with an annealing temperature of 59°C. The sequencing was performed according to the PyroMark Q24 Advanced and PyroMark Q24 Advanced CpG Reagents Handbook (Qiagen), and run on a PyroMark Q24 system. Four individuals from the original study (ID 003, 005, 008, and 009) were reanalyzed using pyrosequencing. The methylation level at each site varied on average 2.5% between the two methods (bisulphite sequencing with a standard curve, and pyrosequencing), and the standard deviation of the difference was 2.8%. (TIF) [file pone.0141805.s002.tif]

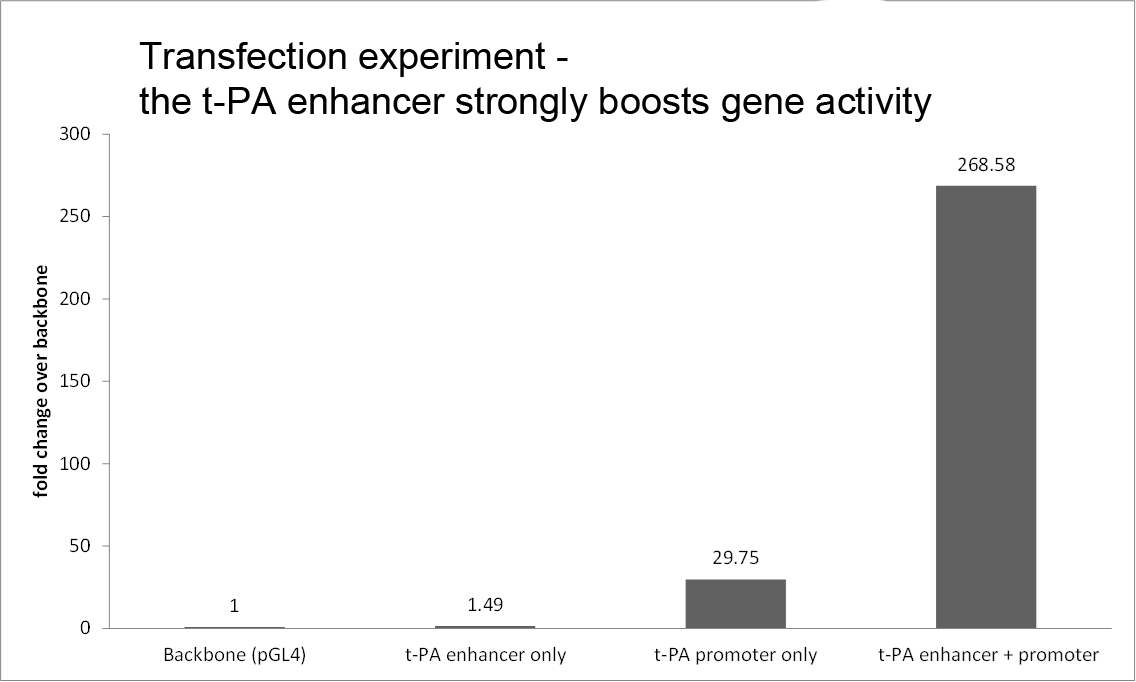

Supplement: S3 Fig — The t-PA proximal promoter and the t-PA enhancer was PCR amplified and cloned into the XhoI/HindIII sites of a firefly luciferase reporter vector (pGL4-Luc, Promega), either each separate, or combined. All constructs were verified by sequencing. Subsequently, the constructs were co-transfected into HT-1080 cells together with the renilla luciferase control vector hRluc/SV40. The luciferase luminescence was measured and normalised to the renilla signal. Fold change of normalised signal is displayed in the figure. Constructs including both promoter and enhancer showed a 9-fold increase in reporter activity compared to constructs containing the promoter alone (p < 0.001, student’s t-test). (TIF) [file pone.0141805.s003.tif]
