# Supplementary material for: Dynamic Enhancer Methylation - A Previously Unrecognized Switch for Tissue-Type Plasminogen Activator Expression
Source: PLoS One. 2015 Oct 28;10(10):e0141805. doi: 10.1371/journal.pone.0141805 (PMC4625093; doi:10.1371/journal.pone.0141805)
Supplement: S1 Table — The methylation average and the change in methylation in the t-PA enhancer (in percent) for all 11 individuals, as well as fold change of t-PA expression (normalised to GUSB) for seven of the individuals (ID005-011). (DOCX) [file pone.0141805.s004.docx]

|  | **Methylation average (%)** | | | **Methylation change (%)** | | **Fold change t-PA** | |
| --- | --- | --- | --- | --- | --- | --- | --- |
|  | **primary** | **p.0** | **p.4** | **p.0** | **p.4** | **p.0** | **p.4** |
| **ID001** | 44 | 24 | 2 | 46 | 96 |  |  |
| **ID002** | 48 | 36 | 2 | 24 | 96 |  |  |
| **ID003** | 58 | 49 | 0 | 15 | 100 |  |  |
| **ID004** | 21 | 4 | 1 | 80 | 95 |  |  |
| **ID005** | 26 | 14 | 1 | 48 | 96 | 19 | 13 |
| **ID006** | 39 | 31 | 4 | 21 | 90 | 5 | 7 |
| **ID007** | 19 | 8 | 1 | 58 | 93 | 30 | 10 |
| **ID008** | 25 | 12 | 0 | 52 | 99 | 7 | 6 |
| **ID009** | 45 | 35 | 6 | 22 | 87 | 4 | 7 |
| **ID010** | 18 | 7 | 0 | 60 | 100 | 22 | 9 |
| **ID011** | 20 | 12 | 1 | 37 | 94 | 17 | 7 |
